# Sustained, Long-Term Maintenance of Remission with Single-Agent Veliparib in Recurrent Triple-Negative Breast Cancer

**DOI:** 10.7759/cureus.1424

**Published:** 2017-07-04

**Authors:** Karthik Kota, Shannon Puhalla

**Affiliations:** 1 Internal Medicine, UPMC Presbyterian; 2 Division of Hematology/Oncology, Magee Women's Hospital of UPMC

**Keywords:** veliparib, parp inhibitor, extraordinary responder, triple-negative breast cancer

## Abstract

Triple-negative breast cancers (TNBC) have poorer outcomes than hormone positive or human epidermal growth factor receptor 2 (HER2)-positive breast cancers, with chemotherapy being the usual standard of care. Veliparib, a poly ADP-ribose polymerase (PARP) inhibitor, has been studied in both breast cancer susceptibility genes 1 and 2 (BRCA)-mutation related and sporadic cancers as a single agent and in combination with chemotherapy. Here, we describe a patient whose metastatic recurrence of TNBC was treated with combination chemotherapy and veliparib followed by maintenance single-therapy veliparib.

## Introduction

Triple-negative breast cancers (TNBC) is defined by the absence of both hormone receptors (estrogen and progesterone) and HER2; as a result, the standard of care for such disease is chemotherapy. Although some TNBCs can respond well to chemotherapy, those cancers that respond poorly to chemotherapy have a high risk of recurrence, particularly in the first three years of therapy and usually to the brain and viscera [1].

The poly ADP-ribose polymerase (PARP) family of proteins is involved in DNA repair, particularly in the base-excision repair pathway for single-strand breaks. Veliparib is an oral inhibitor of PARP1 and PARP2 and is effective against cancer cells via the concept of synthetic lethality (i.e., two conditions that would not cause cell death independently but would when combined). The first condition is usually some type of prior deoxyribonucleic acid (DNA) repair pathway deficit (e.g., a BRCA mutation); in this case, the tumor’s second mutation would allow for selective targeting by the PARP inhibitor, sparing non-mutated cells and potentially avoiding chemotherapy and radiation therapy. Fundamental to this concept is the idea that tumor cells with high mitotic rates can withstand only so much DNA damage before entering mitosis; more than this, and they undergo cell death. Sensitivity to PARP inhibition increases with upregulation of baseline levels of DNA repair proteins (and, conversely, upregulation of DNA repair pathways can limit PARP inhibition effectiveness in models, such as small cell lung cancer). Thus, to potentiate the effects of synthetic lethality, veliparib can be given with DNA-damaging chemotherapy agents like carboplatin [2-4].

## Case presentation

The patient is a 37-year-old woman who presented on December 15, 2008 after noticing a palpable left inferior breast mass.  Initial ultrasound was inconclusive, but repeat ultrasound two weeks later led to a biopsy showing triple-negative infiltrating ductal breast carcinoma, Grade 3, and a Ki-67 of 80%. Follow-up imaging over the first two weeks of 2009 revealed lateral left breast and left axillary lymphadenopathy but no metastases by computed tomography (CT) by chest/abdomen/pelvis or by bone scan. She had a strong family history of breast cancer (mother with breast cancer at 43 and relapse at 56 and a sister with Stage 0 ductal carcinoma in situ at 41) but was BRCA negative. She was enrolled in a clinical trial and underwent four cycles of neoadjuvant capecitabine/docetaxel/bevacizumab (January 22 to March 26, 2009) and four cycles of neoadjuvant doxorubicin/cyclophosphamide/bevacizumab therapy (April 16 to June 23, 2009) before undergoing a bilateral mastectomy with lymph node dissection on July 24, 2009. At the time of surgery, she was found to have a pathologic complete response. Her trial concluded with 18 cycles of adjuvant bevacizumab (September 15, 2009 to March 15, 2010). Her only complication was premature ventricular contractions thought to be secondary to chemotherapy-induced menopause (negative electrocardiograms and echocardiography) and was managed successfully with metoprolol.

**Figure 1:**
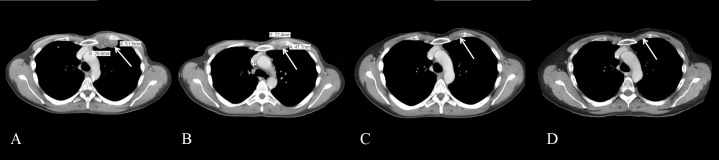
CT Chest with Intravenous Contrast Over Time Images taken three weeks before (A), two months into (B), five months into (C), and nearly six years (D) into treatment for metastatic disease, showing resolution of left chest wall mass

On July 7, 2010, however, during routine follow-up CT scans, CM was found to have recurrent left chest wall and lymph node-associated masses (largest 5.2 x 3 cm) and several bilateral lung nodules (largest 1.5 x 1.7 cm in left upper lobe). On July 27, 2010, the patient began a clinical trial, combining chemotherapy (carboplatin/paclitaxel) with veliparib. By the time of her next scans on October 1, 2010, the patient’s chest CT showed a marked decrease in her anterior chest wall mass, all pulmonary nodules, and all lymph nodes (“noted in records as being a near complete response”) (Figure 1). After nearly a year, the patient stopped the chemotherapy on June 20, 2011 due to CT scans continuing to show stable, minimal disease. She continued on twice-daily single-agent veliparib, with monthly lab tests and CT scans every three months; her course was complicated only by bilateral upper extremity paresthesias without pain or functional deficit. On November 21, 2016, the patient had blood drawn to try to detect circulating cell-free DNA (cfDNA) with the Guardant360 kit (Guardant Health, Inc., Redwood City, CA) with no detectable cfDNA across its 73 detectable genes (National Center for Biotechnology Information (NCBI) Genetic Testing Registry (GTR) ID GTR000527948.4). On April 3, 2017, the patient had expanded germline testing with the myRisk® Hereditary Cancer test (Myriad Genetics, Salt Lake City, UT) (NCBI GTR000530028.2); all results were negative for mutations, deletions, or duplications. No metastatic tumor testing was ever performed due to lack of biopsy. The patient has been clinically stable up to the present day with minimal, tiny lung nodules.

## Discussion

In the metastatic setting, veliparib has been studied in combination with chemotherapy agents to potentiate its effects, or given as a single agent. While there was encouraging activity in the early setting in combination with chemotherapy [5], in the randomized setting, recent results have been disappointing [6]. In patients with BRCA-mutations, veliparib with chemotherapy significantly improved response rates but not progression-free survival; Phase III data are pending (NCT01506609).

The patient has no identifiable DNA repair pathway mutation of her tumor thus far but responded very well to single-agent veliparib after combined carboplatin/paclitaxel/veliparib. One reason for this might be that a DNA repair pathway that was not tested so far is part of the patient’s metastatic disease. As an example, inhibition of the phosphoinositide 3-kinase (PI3K) pathway in TNBC cells can suppress homologous recombination, the pathway by which BRCA allows PARP inhibitors to be effective; addition of GDC-0980 (a dual PI3K-mammalian target of rapamycin inhibitor) to veliparib and carboplatin in BRCA wild-type TNBC cell lines has been shown to increase apoptosis [7]. In general, treatment options for TNBC are limited, and few have sustained response > 2 years [8].

Unfortunately, there wasn’t any tumor sample available for genomic analysis, and there was not any detectable circulating tumor DNA to look for additional mutations that could underlie the patient’s sensitivity to these agents. The patient is one of the rare, sustained (near) complete responders in TNBC. Interestingly, given a pathologic complete response in the neoadjuvant setting and an unclear understanding of the precise mechanisms underlying why her tumor responded so well to chemotherapy twice, her case does highlight that there are some patients who will do remarkably well, even in the metastatic setting with TNBC.

## Conclusions

TNBC is usually treated with chemotherapy and generally carries a poor prognosis, given lower response rates to treatment and a higher risk of recurrence. PARP inhibitors, such as veliparib, are currently under clinical trials to treat TNBC, though they usually show the benefit of objective response (without noted improvements in progression-free survival, thus far) in patients with a prior DNA repair pathway mutation, such as BRCA. Our patient had a complete pathologic response to her initial cancer, then a near complete pathologic response to combination chemotherapy (carboplatin/paclitaxel) and veliparib, and has had sustained remission for nearly six years on single-agent veliparib. Although further genetic testing may elucidate a precise mechanism to veliparib's efficacy in this patient, given her previous remarkable responses to treatment, she may also simply be one of the few patients who do well with TNBC, even in the metastatic setting
